# Trends in Media Coverage During the Monkeypox Outbreak: Content Analysis

**DOI:** 10.2196/45787

**Published:** 2023-06-19

**Authors:** Mio Kato, Fumi Yoshimatsu, Tomoya Saito

**Affiliations:** 1 Center for Emergency Preparedness and Response National Institute of Infectious Diseases Tokyo Japan

**Keywords:** risk perception, protection motivation theory, agenda setting, news media, media, infectious disease, monkeypox

## Introduction

Monkeypox has been spreading in nonendemic countries since May 2022 [1]. The media significantly influences how people react to the spread of emerging and reemerging infectious diseases [2]. Protective motivation theory (PMT) posits that people protect themselves using perceptions from 4 cognitive appraisals: perceived severity, perceived vulnerability, outcome efficaciousness, and self-efficacy [3]. We investigated whether news reports contributed to these 4 PMT components to understand the gaps in people’s health information needs.

## Methods

### Overview

We investigated media coverage by selecting Japanese-language articles with “monkeypox” in the headline published between May 7, 2022 (Japan Standard Time), when the outbreak was reported in the United Kingdom, and July 25, 2022, the day after Japan’s first reported case [4]. We searched 14 media websites using the search term “monkeypox”: overseas television networks (BBC Japan and CNN Japan), Japanese television networks (ANN, NHK, and NTV); overseas newswires (Reuters and Agence France-Presse), Japanese newswires (Kyodo and Jiji), and Japanese newspapers (Asahi, Mainichi, Nikkei, Sankei, and Yomiuri). Materials were excluded if they were videos, contained photos only, or were stock information. The content analysis counted the occurrence of terms appealing to fear (eg, number of infected persons, an increasing number of infected persons and deaths), terms appealing to coping (eg, low risk of infection for the public), and neutral terms (eg, transmission, symptoms, prevention, and treatment).

Next, we used a Boolean search of the Meltwater database using the keywords “monkeypox” and “?” to retrieve tweets posted between May 19 and June 6, 2022. We used Meltwater’s Twitter data because Twitter’s open access policy via its application programming interface (API) has become increasingly restricted [5].

### Ethical Considerations

No ethical approval or informed consent was required since the Twitter data contained no personal information.

## Results

To categorize the volume trends in media coverage, we divided the timeframe into three periods: (1) May 19-28, 2022, which corresponds to the initial phase of outbreak reporting in Europe and the United States; (2) June 15-16, 2022, marked as the judgment period; and (3) June 22-29, 2022, which represents the reporting period for the outbreak in Asia.

Table 1 shows the number and percentage of fear and coping appraisals and neutral information occurrences in the articles during each period. During the second period, reports noted that an emergency committee acting as an advisory body for the World Health Organization (WHO) Director-General would convene to discuss whether monkeypox should be declared a Public Health Emergency of International Concern (PHEIC) [6]. During the third period, reports noted outbreaks in countries geographically close to Japan, specifically, South Korea, Singapore, and Taiwan, and that the WHO had decided not to declare a PHEIC. A high percentage of fear appraisals was reported across all periods, while the number of coping appraisals was substantially lower.

**Figure 1:**
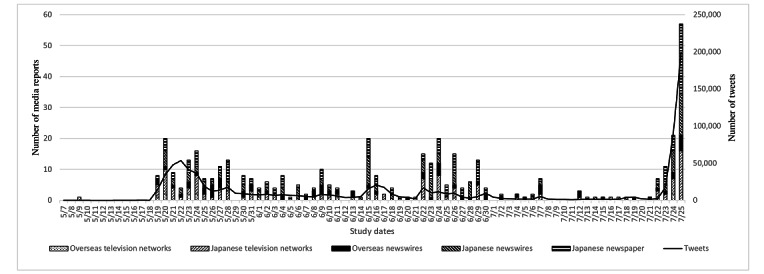
News media coverage and tweets (N=904,252) from May 7 to July 25, 2022. May 7: first case reported by the UK Health Security Agency; July 24: first case reported in Japan. Media consulted include overseas television networks (BBC Japan and CNN Japan), Japanese television networks (ANN, NHK, and NTV), overseas newswires (Reuters and Agence France-Presse), Japanese newswires (Kyodo and Jiji), and Japanese newspapers (Asahi, Mainichi, Nikkei, Sankei, and Yomiuri).

Figure 1 shows the number of articles and tweets by media category chronologically. The graph presents a waveform that corresponds roughly to the first period of media coverage but not to the amount of coverage in the second or third period. For example, the topics that appeared initially from May 19 to June 6, 2022 (N=69,621 tweets) were as follows: “about monkeypox as an infectious disease” (n=38,988, 56%), “vaccine” (n=23,671, 34%), “misinformation” (n=3481, 5%), “measures” (n=2785, 4%), and “concern about economic shutdown” (n=696, 1%).

**Table 1:** Fear appraisals, coping appraisals, and neutral information in Japanese-language articles on monkeypox.

Period	Date and description	Count, n	Fear appraisal^a^, n (%)	Coping appraisal^b^, n (%)	Neutral information^c^, n (%)
1	May 19-28, 2022: initial phase of outbreak reporting in Europe and the United States	108	103 (95.4)	21 (19.4)	77 (71.3)
2	June 15-16, 2022: judgment period	28	26 (92.9)	0 (0)	17 (60.7)
3	June 22-29, 2022: reporting period for the outbreak in Asia	90	84 (93.3)	3 (3.3)	50 (55.6)

^a^Fear appraisal: number of infected persons and increases in the number of infected persons and deaths.

^b^Coping appraisal: low risk of infection for the general public; not contagious like COVID‑19.

^c^Neutral information: human-to-human transmission, symptoms, prevention (vaccination), and treatment.

## Discussion

We found significantly fewer coping appraisals than fear appraisals in the articles included in this content analysis. However, according to our content analysis of tweets, 56% of citizens (38903/69621) wanted neutral information regarding basic facts about infectious diseases, treatment, vaccines, and other prevention methods.

One limitation of this study is that only major news media were included in the analysis; we acknowledge that scientific and medical media may have published different content. However, the impact is significant because the public consumes information via the major media targeted in this study and disseminates it on social networking platforms. Therefore, confirming the content of such media is essential.

We recommend that crisis management teams should provide easy-to-understand information enabling the media to disseminate basic information on infectious diseases and messages that lead to coping appraisals.

## Abbreviations

PHEIC: Public Health Emergency of International Concern
PMT: protective motivation theory
WHO: World Health Organization
